# Gender difference in relationship between body mass index and development of chronic kidney disease

**DOI:** 10.1186/1756-0500-6-463

**Published:** 2013-11-13

**Authors:** Hiroshi Komura, Ikuo Nomura, Kazuo Kitamura, Kenji Kuwasako, Johji Kato

**Affiliations:** 1 Department of Internal Medicine, Circulatory and Body Fluid Regulation, Faculty of Medicine, Kiyotake, Miyazaki 889-1692, Japan; 2 Frontier Science Research Center, University of Miyazaki, 5200 Kihara, Kiyotake, Miyazaki 889-1692, Japan

**Keywords:** Chronic kidney disease, Body mass index, Obesity, General population

## Abstract

**Background:**

An epidemiological approach to preventing the development or progression of chronic kidney disease (CKD) is necessary, while few effective preventive measures are currently available. We conducted a community-based, cohort study to identify the factors associated with the development of CKD in the general population.

**Methods:**

We examined 1876 local residents of a Japanese community who had an annual health check-up and, of those, 1506 residents judged not to have CKD (473 men and 1033 women) were followed for the development of CKD over 10 years.

**Results:**

The numbers of male and female residents who developed CKD during the follow-up period were 167 (35.3%) and 299 (28.9%), respectively. As compared to those without CKD development, the residents who developed CKD were older, and had a higher body mass index (BMI), systolic blood pressure, and creatinine in both genders. The rate of CKD development in obese female residents was higher than in non-obese women, but such a difference was not noted in male residents. In addition to age and serum creatinine, we identified BMI as an independently significant factor for the development of CKD in women, but not in men.

**Conclusions:**

Increased BMI is a significant risk factor for the development of CKD in women, and there seems to be a gender difference in the association between increased BMI and the development of CKD in the general population.

## Background

Chronic kidney disease (CKD) is defined as either structural and/or functional abnormality of the kidney or a reduced glomerular filtration rate (GFR) to <60 ml/min/1.73 m^2^[1,2]. CKD has recently been recognized as a challenging public health issue not only because the number of patients with end-stage renal disease (ESRD) is steadily increasing in developed countries including Japan, but also because epidemiological studies have shown that CKD is a significant risk factor for cardiovascular disease [3-5]. For example, the number of ESRD patients in Japan is currently some 300,000, and this has become an economic burden for the health care system [6]. On the other hand, it was shown in a Japanese community by a prospective study that ischemic heart disease and cerebral infarction are more frequent in male and female residents with CKD, respectively, than in those without [5]. In order to prevent or reduce the development of CKD at a community level, it is essential to identify or specify the causes of CKD. Possible factors shown to be involved in the development of CKD are age, hypertension, impaired glucose tolerance or diabetes mellitus, dyslipidemia and obesity [7-16]; however, the detailed mechanisms remains to be clarified, and we currently have few effective countermeasures to prevent the development or progression of CKD at a community level. According to our previous cross-sectional study, body mass index (BMI) was found to be an independently, significant factor for CKD, in addition to age, in male and female residents of the Kiyotake area, a Japanese community [17]. In the present study, we followed the local residents of that area, who had been judged not to have CKD, for 10 years to identify the factors associated with future development of CKD in a general population.

## Methods

In this study, we examined local residents in the Kiyotake area, Miyazaki, Japan, who had an annual health check-up in October 1999, responding to the recommendations made by the municipal government for those of 40 years or older. Upon visiting the community center of Kiyotake town, blood pressure was measured with an oscillometric automatic device (BP-103iII; Colin, Tokyo, Japan) in a sitting position by experienced nurses. Then, urine was collected and blood was drawn from an antecubital vein. Serum lipid and glucose levels were measured by an automatic analyzer (AU2700; OLYMPUS, Tokyo, Japan) with serum creatinine levels determined by an enzymatic method. GFRs were calculated with the formula of the Japanese Society of \Nephrology: 194 × serum creatinine^- 1.094^ × age^-0.287^ ml/min/1.73 m^2^, further multiplied by 0.739 for women [18]. Residents were judged to have CKD when the estimated GFR was <60 ml/min/1.73 m^2^ or spot urine protein determined by dipstick measurement was ≥ +1 (30 mg/dl). Obesity was defined as BMI ≥25 kg/m^2^, according to the criteria of the Japan Society for the Study of Obesity [19]. In this health check-up program, 1876 residents (615 men and 1261 women; 58.7 ± 11.6 years; mean ± S.D.) visited during the time period mentioned above. Of those receiving the check-up in 1999, 1506 residents, who were judged not to have CKD (473 men and 1033 women; 58.2 ± 11.0 years), were followed up for the development of CKD over 10 years at the annual health check-ups held by the municipal government. The residents receiving the annual health check-ups had been notified of the results by mail every year.

This study was approved by the Review Committee of Cooperative and Commissioned Research and the Ethics Committee of the University of Miyazaki Faculty of Medicine. The check-up programs are carried out by the municipal government according to Japanese law. In the present study, we used the database of the municipal government, from which it was impossible for us to identify individual residents. Therefore, written informed consent was not obtained individually, but instead, the local residents were informed of this study via a local newspaper issued by the municipal government, as required by the Ethics Committee.

All the data were analyzed with IBM SPSS software version 20.0 (IBM, Armonk, NY, USA). Two groups were compared by the unpaired *t*-test or chi-squared test. The Kaplan-Meier method and log rank test were used to see differences in the development of CKD between obese and non-obese residents. The relationships between the development of CKD and the other parameters were tested by Cox proportional hazard models. All data are expressed as the means ± S.D. and P <0.05 was considered to be significant.

## Results

**Figure 1:**
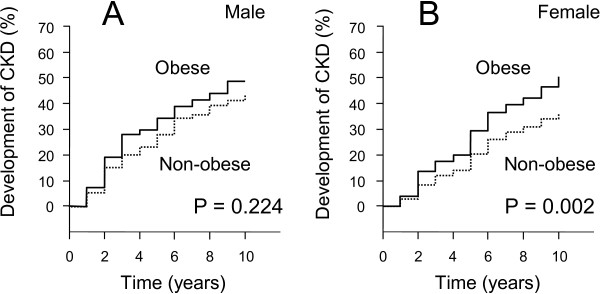
Kaplan-Meier analyses for the development of CKD in male (A) and female (B) residents.

Table 1 shows the basal profiles of the male and female residents examined in this study. As shown, 167 male and 299 female residents were found to have developed CKD during 1999 to 2009, the diagnosis of which was made by estimated GFR <60 ml/min/1.73 m^2^ in 268 residents, by dipstick proteinuria ≥ +1 in 188 or by both in 10. The development of CKD in male residents (35.3%) was more frequent (P <0.05) than in female residents (28.9%). The residents who had developed CKD were older, showing higher systolic blood pressure and serum creatinine than those without CKD development, in both genders. Additionally, fasting blood glucose in men and aspartate aminotransferase (AST) in women were higher in those with CKD development. BMI in male and female residents who developed CKD was higher, and obesity was significantly more prevalent in women than in those who did not develop CKD. In the Kaplan-Meier analysis (Figure 1), no significant difference in CKD development was noted between obese and non-obese male (A), but CKD occurred more frequently in obese than in non-obese women (B). Comparable with this, there was no difference in newly developed proteinuria between obese and non-obese male residents (16.8 vs. 16.7%), while development of proteinuria during the follow-up period was more frequent in obese than in non-obese women (20.2 vs. 11.7%, P < 0.01).

**Table 1:** Basal profiles of male and female residents with or without development of CKD

	**Men**		**Women**	
**Development of CKD**	**(−)**	**(+)**	**(−)**	**(+)**
n	306	167	734	299
Age (years)	58.0 ±11.5	64.6 ± 8.9**	56.2 ± 10.6	59.6 ± 11.1**
Body mass index (kg/m^2^)	22.8 ± 2.9	23.4 ± 3.0*	22.1 ± 3.0	23.0 ± 3.2**
Obesity (%)	21.6	28.1	16.5	24.1**
Systolic blood pressure (mmHg)	124 ± 18	132 ± 18**	122 ± 17	125 ± 18**
Diastolic blood pressure (mmHg)	76 ± 11	78 ± 10*	73 ± 10	74 ± 10
Total cholesterol (mg/dL)	195 ± 32	194 ± 34	205 ± 33	207 ± 34
HDL-cholesterol (mg/dL)	57 ± 15	56 ± 15	64 ± 15	62 ± 15
Triglyceride (mg/dL)	106 ± 73	103 ± 52	88 ± 45	94 ± 56
Fasting blood glucose (mg/dL)	93 ± 15	97 ± 17*	90 ± 12	90 ± 10
AST (IU/L)	26 ± 8	27 ± 11	22 ± 7	24 ± 9*
ALT (IU/L)	24 ± 13	26 ± 22	18 ± 10	20 ± 17
γ-GTP (IU/L)	43 ± 50	58 ± 96	21 ± 22	22 ± 21
Creatinine (mg/dl)	0.71 ± 0.09	0.78 ± 0.12**	0.51 ± 0.08	0.59 ± 0.09**

AST, aspartate aminotransferase; ALT, alanine aminotransferase; GTP, glutamyl transpeptidase; means ± S.D.; *P < 0.05, **P < 0.01, vs. without development of CKD.

Next, we analyzed the development of CKD by Cox proportional hazard models with single or multiple covariates, as shown in Tables 2 and 3. In univariate analysis, age, systolic and diastolic blood pressure, aspartate aminotransferase (AST), and creatinine levels were found to be significant covariates for CKD development in both genders. Also shown to be significant were fasting blood glucose levels in male residents and triglyceride in female residents. BMI was judged to be a significant factor in female residents but not in male residents. We then conducted multivariate analyses, where clinical parameters were used as independent covariates if they were significantly different between residents with and without CKD development (Table 1) or judged to be a significant factor by univariate analyses. As shown in the right columns of Tables 2 and 3, age and serum creatinine were significant in both genders, but similar to univariate analysis, BMI was extracted as a significant factor only in female residents.

**Table 2:** Identification of factors associated with development of CKD in male subjects

**Male**	**Univariate**	**Multivariate**
	**RR (95% CI)**	**RR (95% CI)**
Age (years)	1.058 (1.041-1.075)**	1.051 (1.032-1.070)**
Body mass index (kg/m^2^)	1.038 (0.987-1.092)	1.035 (0.978-1.096)
Systolic blood pressure (mmHg)	1.020 (1.012-1.029)**	1.008 (0.996-1.021)
Diastolic blood pressure (mmHg)	1.019 (1.004-1.034)*	1.002 (0.981-1.023)
Total cholesterol (mg/dL)	0.999 (0.995-1.004)	
HDL-cholesterol (mg/dL)	0.996 (0.986-1.006)	
Triglyceride (mg/dL)	0.999 (0.997-1.002)	
Fasting blood glucose (mg/dL)	1.012 (1.004-1.019)**	1.009 (0.999-1.018)
AST (IU/L)	1.016 (1.001-1.031)*	1.001 (0.979-1.023)
ALT (IU/L)	1.005 (0.996-1.013)	
γ-GTP (IU/L)	1.002 (1.000-1.003)*	1.002 (1.000-1.004)
Creatinine (mg/dl)	160 (33–785)**	101 (20–522)**

RR, risk ratio; AST, aspartate aminotransferase; ALT, alanine aminotransferase; GTP, glutamyl transpeptidase; *P < 0.05, **P < 0.01.

**Table 3:** Identification of factors associated with development of CKD in female subjects

**Female**	**Univariate**	**Multivariate**
	**RR (95% CI)**	**RR (95% CI)**
Age (years)	1.034 (1.022-1.046)**	1.030 (1.017-1.044)**
Body mass index (kg/m^2^)	1.080 (1.042-1.119)**	1.048 (1.008-1.089)*
Systolic blood pressure (mmHg)	1.012 (1.005-1.018)**	1.008 (0.998-1.019)
Diastolic blood pressure (mmHg)	1.012 (1.000-1.023)*	0.995 (0.978-1.012)
Total cholesterol (mg/dL)	1.002 (0.999-1.005)	
HDL-cholesterol (mg/dL)	0.993 (0.985-1.000)	
Triglyceride (mg/dL)	1.002 (1.000-1.004)*	0.999 (0.997-1.002)
Fasting blood glucose (mg/dL)	1.005 (0.996-1.014)	
AST (IU/L)	1.015 (1.002-1.028)*	1.003 (0.988-1.018)
ALT (IU/L)	1.006 (0.999-1.014)	
γ-GTP (IU/L)	1.001 (0.997-1.006)	
Creatinine (mg/dl)	6290 (1472–26882)**	6870 (1549–30470)**

RR, risk ratio; AST, aspartate aminotransferase; ALT, alanine aminotransferase; GTP, glutamyl transpeptidase; *P < 0.05, **P < 0.01.

## Discussion

Chronic kidney disease (CKD) has recently been recognized not only as a risk factor of ESRD, but also of cardiovascular disease, which is a leading cause of death in developed countries [3-5]. An interventional approach is necessary to prevent the development or progression of CKD, while a number of factors have been reported to be involved in the development of CKD and currently there are few specific countermeasures against CKD at community levels. Obesity has been shown to be an important factor associated with CKD [10,14,16,20,21], and indeed, according to our previous, community-based, cross-sectional study [17], BMI was found to be significant for CKD in the Kiyotake area, a Japanese community, in both genders. We therefore conducted this cohort, 10-year follow-up study with local residents without CKD in the same area, and found that an increase in BMI was independently and significantly associated with the development of CKD only in women.

It has been suggested that a number of factors are associated with the development of CKD: these are male gender, age, hypertension, impaired glucose tolerance or diabetes mellitus, dyslipidemia, obesity, and smoking [7-16]. Accordant with those reports, the development of CKD was more frequent in male residents than in female residents, and age was determined to be a significant factor of CKD development in both genders in the present study. Obesity is closely associated with hypertension, diabetes mellitus, and dyslipidemia, all of which are detrimental factors to renal function [7-12]. Indeed, systolic and diastolic blood pressure levels were significant for the development of CKD in both genders in univariate analyses in the present study. Also found to be significant were the levels of fasting blood glucose in men and those of triglycerides in women. Meanwhile, obesity has recently been shown to be a risk factor for the development or progression of CKD, which is independent of the other risks mentioned above [13,16,22]. Possible mechanisms of this obesity-induced renal damage are fat tissue-derived factors detrimental to renal function, such as tumor necrosis factor-α, interleukin-6 and plasminogen activator inhibitor-1 [22].

An interesting finding of the present study was a gender difference in the relationship between the development of CKD and obesity or BMI. As shown in the basal profiles of the study subjects (Table 1), BMI in the residents with CKD development was higher than in those without in both genders; however, significant associations between CKD development and BMI or obesity were found only in women by Kaplan-Meier and univariate or multivariate analyses. A similar finding was observed by Yamagata et al. [15], who reported that obesity was a significant factor for CKD development only in women in a Japanese community, although no further data, including BMI, were available in their paper. We examined male and female obese residents with basal or metabolic parameters possibly involved in the development of CKD, but failed to find any clear difference between these two groups (data not shown); therefore, there seem to be no clear explanations for the gender difference in CKD development observed in the present study. This important point is discussed below in comparison with the results from cohort studies of subjects without CKD and from cross-sectional studies.

We previously conducted a cross-sectional study of local residents of the same community as examined in the present study, and found a significant relationship between CKD and BMI, which was independent of age and other cardiovascular risk factors in both genders [17]. Meanwhile, Ishizaka et al. and Shankar et al. performed similar cross-sectional community-based studies, showing significant relationships between CKD and BMI in men but not in women, suggesting the susceptibility of obese men to CKD in comparison with obese women [20,21]. The findings of these studies appear to contradict those of the present study, where obese women were prone to CKD compared with non-obese women, but such a difference was not noted in men. Possible explanations for this discrepancy are as follows. First, the present study is a cohort study where subjects without CKD were followed up over 10 years, hence, both the study design and the study subjects were essentially different. Second, the age of the subjects may be a factor: the mean age of the subjects in those studies was around 60 years, while in the present study, residents of a similar age range were followed over 10 years. A relationship between CKD and obesity might have been found in men if younger subjects had been followed for a certain period of time. In an attempt to prove this, we analyzed the data of male residents younger than 60 years, but the number of subjects was relatively small (n = 189) and no significant difference was noted in CKD development between obese and non-obese men; therefore, this hypothesis needs to be tested by future studies.

Lastly, we need to mention the limitations of the present study. First, there might have been a selection bias because the local residents examined in this study were those who received a health check-up responding to the recommendations made by the municipal government. For example, because the present subjects had relatively lower BMI with smaller standard deviations than in other studies [12,21], the remainder of the general population might have different risk factors for CKD development. Additionally, a type 2 statistical error cannot completely be excluded from the gender difference: the number of male residents examined was smaller than that of female residents, while the Kaplan-Meier curves of CKD development differed between male and female residents with substantially different p values (Figure 1). Second, the subjects examined in this study were ethnically Japanese; therefore, caution should be taken when the present findings are discussed in comparison with other races or ethnic groups. Third, no data were available regarding whether the study subjects had hypertension or diabetes mellitus, important factors in CKD development, at enrollment in this study; instead, the data were adjusted by blood pressure and fasting blood glucose levels.

## Conclusions

Increased BMI was significantly associated with the development of CKD in female residents in the general population, while such a relationship was unclear in male residents, suggesting a possible difference in susceptibility to CKD associated with obesity. Interventional studies or approaches are warranted to see the effect of body weight reduction on the development of CKD at community levels.

## Abbreviations

CKD: Chronic kidney disease; BMI: Body mass index; ESRD: End-stage renal disease; GFR: Glomerular filtration rate; AST: Aspartate aminotransferase; ALT: Alanine aminotransferase; GTP: Glutamyl transpeptidase.

## Competing interests

The authors declare that they have no competing interests.

## Authors’ contributions

HK, IN, and JK carried out the regular health check-ups for the residents and collection of the data. KK, KK, and JK analyzed the collected data statistically. All authors have read and approved the final manuscript.

## References

[B1] National Kidney Foundation K/DOQI clinical practice guidelines for chronic kidney disease: evaluation, classification, and stratification Am J Kidney Dis 2002 39 2 Suppl 1 S1 S266 11904577

[B2] Levey AS Coresh J Balk E Kausz AT Levin A Steffes MW Hogg RJ Perrone RD Lau J Eknoyan G National Kidney Foundation practice guidelines for chronic kidney disease: evaluation, classification, and stratification Ann Intern Med 2003 139 137 147 10.7326/0003-4819-139-2-200307150-00013 12859163

[B3] Sarnak MJ Levey AS Schoolwerth AC Coresh J Culleton B Hamm LL McCullough PA Kasiske BL Kelepouris E Klag MJ Parfrey P Pfeffer M Raij L Spinosa DJ Wilson PW Kidney disease as a risk factor for development of cardiovascular disease: a statement from the American Heart Association Councils on kidney in cardiovascular disease, high blood pressure research, clinical cardiology, and epidemiology and prevention Hypertension 2003 42 1050 1065 10.1161/01.HYP.0000102971.85504.7c 14604997

[B4] Go AS Chertow GM Fan D McCulloch CE Hsu CY Chronic kidney disease and the risks of death, cardiovascular events, and hospitalization N Engl J Med 2004 351 1296 1305 10.1056/NEJMoa041031 15385656

[B5] Ninomiya T Kiyohara Y Kubo M Tanizaki Y Doi Y Okubo K Wakugawa Y Hata J Oishi Y Shikata K Yonemoto K Hirakata H Iida M Chronic kidney disease and cardiovascular disease in a general Japanese population: the Hisayama study Kidney Int 2005 68 228 236 10.1111/j.1523-1755.2005.00397.x 15954912

[B6] The Japanese Society of Nephrology Clinical practice guidebook for diagnosis and treatment of chronic kidney disease 2012 Jpn J Nephrol 2012 54 1031 1189

[B7] Klag MJ Whelton PK Randall BL Neaton JD Brancati FL Ford CE Shulman NB Stamler J Blood pressure and end-stage renal disease in men N Engl J Med 1996 334 13 18 10.1056/NEJM199601043340103 7494564

[B8] Iseki K Iseki C Ikemiya Y Kinjo K Takishita S Risk of developing low glomerular filtration rate or elevated serum creatinine in a screened cohort in Okinawa, Japan Hypertens Res 2007 30 167 174 10.1291/hypres.30.167 17460387

[B9] Chen J Muntner P Hamm LL Jones DW Batuman V Fonseca V Whelton PK He J The metabolic syndrome and chronic kidney disease in U.S. adults Ann Intern Med 2004 140 167 174 10.7326/0003-4819-140-3-200402030-00007 14757614

[B10] Hallan S De Mutsert R Carlsen S Dekker FW Aasarød K Holmen J Obesity, smoking, and physical inactivity as risk factors for CKD: are men more vulnerable? Am J Kidney Dis 2006 47 396 405 10.1053/j.ajkd.2005.11.027 16490617

[B11] Fox CS Larson MG Leip EP Meigs JB Wilson PWF Levy D Glycemic status and development of kidney disease. The Framingham heart study Diabetes Care 2005 28 2436 2440 10.2337/diacare.28.10.2436 16186276

[B12] Tanaka H Shiohira Y Uezu Y Higa A Iseki K Metabolic syndrome and chronic kidney disease in Okinawa, Japan Kidney Int 2006 69 369 374 10.1038/sj.ki.5000050 16408128

[B13] Nguyen S Hsu CY Excess weight as a risk factor for kidney failure Curr Opin Nephrol Hypertens 2007 16 71 76 10.1097/MNH.0b013e32802ef4b6 17293680

[B14] Kramer H Luke A Bidani A Cao G Cooper R McGee D Obesity and prevalent and incident CKD: the hypertension detection and follow-Up program Am J Kidney Dis 2005 46 587 594 10.1053/j.ajkd.2005.06.007 16183412

[B15] Yamagata K Ishida K Sairenchi T Takahashi H Ohba S Shiigai T Narita M Koyama A Risk factors for chronic kidney disease in a community-based population: a 10-year follow-up study Kidney Int 2007 71 159 166 10.1038/sj.ki.5002017 17136030

[B16] Wang Y Chen X Song Y Caballero B Cheskin LJ Association between obesity and kidney disease: a systematic review and meta-analysis Kidney Int 2008 73 19 33 10.1038/sj.ki.5002586 17928825

[B17] Nomura I Kato J Kitamura K Association between body mass index and chronic kidney disease: a population-based, cross-sectional study of a Japanese community Vasc Health Risk Manag 2009 5 315 320 19436662 10.2147/vhrm.s5522 PMC2672451

[B18] Matsuo S Imai E Horio M Yasuda Y Tomita K Nitta K Yamagata K Tomino Y Yokoyama H Hishida A Revised equations for estimated GFR from serum creatinine in Japan Am J Kidney Dis 2009 53 982 992 10.1053/j.ajkd.2008.12.034 19339088

[B19] Examination Committee of Criteria for ‘Obesity Disease’ in Japan, Japan Society for the Study of Obesity New criteria for ‘obesity disease’ in Japan Circ J 2002 66 987 992 10.1253/circj.66.987 12419927

[B20] Ishizaka N Ishizaka Y Toda E Koike K Seki G Nagai R Yamakado M Association between obesity and chronic kidney disease in Japanese: differences in gender and hypertensive status? Hypertens Res 2007 30 1059 1064 10.1291/hypres.30.1059 18250555

[B21] Shankar A Leng C Chia KS Koh D Tai ES Saw SM Lim SC Wong TY Association between body mass index and chronic kidney disease in men and women: population-based study of Malay adults in Singapore Nephrol Dial Transplant 2008 23 1910 1918 10.1093/ndt/gfm878 18156460

[B22] Hunley TE Ma LJ Kon V Scope and mechanisms of obesity-related renal disease Curr Opin Nephrol Hypertens 2010 19 227 234 10.1097/MNH.0b013e3283374c09 20134323 PMC2897176

